# The genetic diversity of commensal *Escherichia coli* strains isolated from non-antimicrobial treated pigs varies according to age group

**DOI:** 10.1371/journal.pone.0178623

**Published:** 2017-05-30

**Authors:** Shahana Ahmed, John E. Olsen, Ana Herrero-Fresno

**Affiliations:** Department of Veterinary and Animal Sciences, Faculty of Health and Medical Sciences, University of Copenhagen, Frederiksberg, Denmark; Wilfrid Laurier University, CANADA

## Abstract

This is the first report on the genetic diversity of commensal *E*. *coli* from pigs reared in an antibiotic free production system and belonging to different age groups. The study investigated the genetic diversity and relationship of 900 randomly collected commensal *E*. *coli* strains from non-antimicrobial treated pigs assigned to five different age groups in a Danish farm. Fifty-two unique REP profiles were detected suggesting a high degree of diversity. The number of strains per pig ranged from two to 13. The highest and the lowest degree of diversity were found in the early weaners group (Shannon diversity index, *H'* of 2.22) and piglets (*H'* of 1.46) respectively. The REP profiles, R1, R7 and R28, were the most frequently observed in all age groups. *E*. *coli* strains representing each REP profile and additional strains associated with the dominant profiles were subjected to PFGE and were assigned to 67 different genotypes. Whole genome sequence analysis of 52 isolates leading to unique REP profiles identified a high level of sequence variation. Six and six strains were assigned to sequence type ST10 and sequence type ST58, respectively. Virulence and antimicrobial resistance genes, as well as, genes associated with mobile genetic elements were commonly found among these commensal *E*. *coli* strains. Interestingly, strains yielding the three most common REP profiles clustered together in the SNPs phylogenetic tree, and such strains may represent the archetypal commensal *E*. *coli* in Danish pigs.

## Introduction

Most *E*. *coli* are harmless inhabitants of the intestines of humans and animals, where they constitute a high proportion of the facultative anaerobic commensal microbiota [1,2]. In swine, piglets acquire intestinal microorganisms from the mother at birth, including *E*. *coli* [3]. *E*. *coli* strains are often used as indicator bacteria in different kind of studies, such as those on antimicrobial resistance and those on genetic diversity of the intestinal microbiota [4]. The population structure of commensal *E*. *coli* is determined by multiple host and environmental factors [1], including antimicrobial treatment, which might significantly influence the genetic structure of the *E*. *coli* population [2].

Emergence of antimicrobial resistance in humans and animals has led to a global public health concern [5]. Denmark is one of the leading pig producing countries in the world, and has a highly intensive pig production. While antimicrobials are commonly used for treatment in this production systems, Denmark has gradually reduced the level of antimicrobial usage in livestock and currently produces pigs using one of the lowest amounts of antimicrobials in Europe when corrected for number of animals produced [6]. As a relatively new development, pig production systems without administration of antimicrobials to the majority of animals are now emerging [7]. The main reason behind is that it is well documented, that use of antimicrobials in livestock may contribute to the selection and spread of antimicrobial resistant bacteria, as well as the genetic elements carrying resistance genes which might have a great impact on human health [8–10]. Antimicrobial treatment has been hypothesized to decrease the genetic diversity of the intestinal microbiota [11]. However, so far, genetic diversity of commensal *E*. *coli* from pigs has only been analyzed in nursery pigs subjected to different kinds of antimicrobial treatments [2]. With the emergence of antimicrobial-free pig production systems, it is now possible to estimate how genetic diversity under intensive pig production conditions without use of antimicrobials differs between different age groups.

In general, the commensal *E*. *coli* microbiota of pigs is poorly characterized, and it has never been systematically investigated which sequence types of *E*. *coli* dominate in the commensal flora in the absence of antimicrobials, nor whether this commensal flora is an important reservoir for virulence and antimicrobial resistance genes. Therefore, the aims of the current study were; i) to investigate the genetic diversity and relationship of *E*. *coli* commensal strains collected from healthy, non-antimicrobial treated pigs belonging to different age groups by using molecular typing methods and, ii) to characterize the strains assigned to the unique profiles identified by a whole genome sequence (WGS) approach.

## Materials and methods

### Collection and preparation of samples

A trained veterinarian collected rectal fecal samples from 20 pigs in a Danish farm, where the animals were raised from birth to slaughter without treatment with antimicrobials. When animal welfare considerations dictated the use of antimicrobials for some pigs, such animals were removed from the antimicrobial-free production system before treatment, and they were not allowed back into this farm. All procedures were carried out in agreement with the Animals Scientific Act and performed under the license and approval of the Danish National Animal Experiment Inspectorate (license no. 2009/561-1675). All samples were obtained in 2015. These pigs belonged to five different age groups (four randomly picked pigs per age group) namely piglets, early weaners, late weaners, finishers, and sows. After being collected, the fecal samples were immediately placed in cooling boxes containing ice bags and sent to the laboratory for analysis the next day. Ten-fold serial dilutions of the fecal samples (10% w/v) were prepared in isotonic saline solution (0.9% NaCl) and plated on MacConkey agar (Oxoid, Thermo Scientific, Roskilde, Denmark) for quantification using the spot method [12]. Plates were incubated overnight at 37°C. Lactose positive, dark red colonies with a diameter >0.5 mm were counted in drops containing between 20 and 80 colonies.

### Bacterial isolation and identification

Forty-five lactose positive, dark-red colonies from each fecal sample (N = 900 colonies) were randomly selected. The isolates were confirmed to be *E*. *coli* using standard biochemical characterization (API-20E; bioMerieux, Ballerup, Denmark).

### REP-PCR genomic fingerprinting

The genomic DNA of *E*. *coli* isolates was extracted by using the boiling lysis method as previously described [13]. The REP-PCR oligonucleotide primers used in this study were *Rep1R-I* (5'-III ICG ICG ICA TCI GGC-3') and *Rep2-I* (5'-ICG ICT TAT CIG GCC TAC-3') [14]. The PCR reaction mix (25 μl) contained 2.5 μl (50 ng) of DNA template, 3.5 μl of each primers (10 μmol 1^−1^ stock), 2.5 μl of Dimethyl Sulfoxide (Sigma-Aldrich, Brøndby, Denmark) and 13 μl of Dream Taq Green DNA Polymerase (Thermo Fisher Scientific, Roskilde, Denmark). The PCR reaction was performed using previously described conditions [2,14]. Sterile milliQ water and genomic DNA of *E*. *coli* K-12 strain W3110 were used as negative and positive control, respectively. PCR products were visualized by electrophoresis in 1.5% agarose gel containing ethidium bromide (Roth Nordic A/S, Frederikssund, Denmark). GeneRuler 100 bp plus DNA ladder (Thermo Fisher Scientific, Roskilde, Denmark) was used as an external reference standard to assign fingerprint profiles. Gels were visualized by Gel Doc 1000 (Bio-Rad Laboratories, Hercules, CA, U.S.A.) using Quantity One image capture software, version 4.2.2.

BioNumerics version 7.6 (Applied Maths, Sint-Martens-Latem, Belgium) was used to analyze REP-PCR DNA fingerprints data. Every gel was normalized using the 100 bp plus DNA ladder, which ranges from 100 to 3000 bp as an external reference standard. DNA fingerprint similarities were calculated using the curve-based Pearson coefficient with 1% optimization, and a dendrogram was generated using the unweighted-pair-group method with arithmetic averages (UPGMA). Clusters were considered at a 60% similarity cut-off [15]. The Shannon diversity index (*H´*) was used to determine the genetic diversity of the *E*. *coli* strains and was calculated according to the following formula [16].

$$H^{\prime }=-\sum_{i=1}^{s}p_{i}\text{ln}p_{i}$$

Where S is the number of unique genotype; *p*_*i*_ is the number of isolates sharing the same genotype [i] over the total number of isolates.

Diversity among age groups was also analyzed with GraphPad Prism 6.1 software by using one-way ANOVA analysis with pair-wise comparison of means and Tukey's multiple comparison test [17]. A P value < 0.05 was considered statistically significant.

### Pulse-field gel electrophoresis (PFGE)

Strains showing unique REP profiles and several strains assigned to the dominant REP profiles were subjected to PFGE performed according to the PulseNet standardized protocol [18]. Briefly, chromosomal DNA embedded in solid agarose plugs was digested with the restriction enzyme XbaI (New England Biolabs, Massachusetts, USA, 20,000 units ml^-1^). The DNA fragments were separated by using 1% agarose gel (SeaKem^®^ gold agarose, Rockland, USA) in 0.5X TBE buffer. CHEF-DR III System (Bio-Rad Laboratories, Hercules, CA, U.S.A.) was used to perform electrophoresis at 14°C for 18 hours with initial switch time 2.2 s and final switch time 54.4 s. *E*. *coli* strain 722-1505-26n EC (with known bands sizes) [19] was used as reference marker.

Also here, BioNumerics version 7.6 (Applied Maths, Sint-Martens-Latem, Belgium) was used to analyze the relatedness of the PFGE fingerprints, and similarities were calculated as described above. A cut off value of 60% was used to establish clusters based on PFGE [20].

### Whole-genome sequence analysis (WGS)

*E*. *coli* isolates yielding unique REP profiles were selected for WGS (N = 52). Genomic DNA (gDNA) was extracted using the DNeasy Blood and Tissue Kit (Qiagen, Hilden, Germany). Concentration of extracted DNA was measured by dsDNA BR Assay Kit with the Qubit^®^ 2 Flurometer (Invitrogen, Paisley, UK). Subsequently, the gDNA was subjected to pair end sequencing in an Illumina MiSeq (Illumina, San Diego, USA). The pair-end sequence reads were assembled in an online tool ‘Assembler’ (Version 1.2) available in Center for Genomic Epidemiology (CGE) (www.cge.cbs.dtu.dk) which applies Velvet algorithm for de novo assembly [21]. The assembled genomes were analyzed in CGE webserver by using a newly developed integrated platform called bacterial analysis pipeline (BAP) for analyzing bacterial WGS Data [22]. BAP enables to identify bacterial species (KmerFinder-2.1), acquired antimicrobial resistance genes (ResFinder-2.1), virulence genes (VirulenceFinder-1.2), multilocus sequence type (MLST-1.6) and replicons of bacterial plasmids (PlasmidFinder-1.2). In addition we used SerotypeFinder 1.1 to identify the serotypes of the *E*. *coli* strains. Phylogenetic relationships were determined based on SNP tree constructed by applying the CSI phylogeny tool available in CGE (CSI Phylogeny 1.4) [23]. *E*. *coli* K12 substrain MG1655 genome (GenBank accession number NC_000913.3) was used as reference sequence, and CGE default parameters were used during SNP analysis. The phylogenetic tree was edited by FigTree version 1.4.3 (http://tree.bio.ed.ac.uk/software/figtree/). The raw sequence reads were submitted to the European Nucleotide Archive (ENA) (http://www.ebi.ac.uk/ena) under a study accession number ‘PRJEB15511’ to further obtain the specific accession number of each genome.

## Results

### Counts of fecal *E*. *coli*

Total average count of coliform in each age group (four pigs per age group) were 8±0.53 log_10_ cfu/g in piglets, 7±0.03 log_10_ cfu/g in early weaners, 7±0.53 log_10_ cfu/g in late weaners, 7±0.48 log_10_ cfu/g in finishers, and 8±0.54 log_10_ cfu/g in sows. No significant differences were observed.

### Genetic diversity and relatedness of *E*. *coli* strains from different age groups

In this study we used REP-PCR to analyze the genetic diversity, since it has previously been shown to have a good discriminatory power [2,24–26]. It is also a simpler method than other molecular typing techniques, which allows handling a large number of strains [2,24]. Furthermore, we used this typing method in order to compare our current results with those obtained in our previous studies on genetic diversity of *E*. *coli* from nursery pigs raised in farms where antimicrobials were administered [2,19].

**Fig 1:**
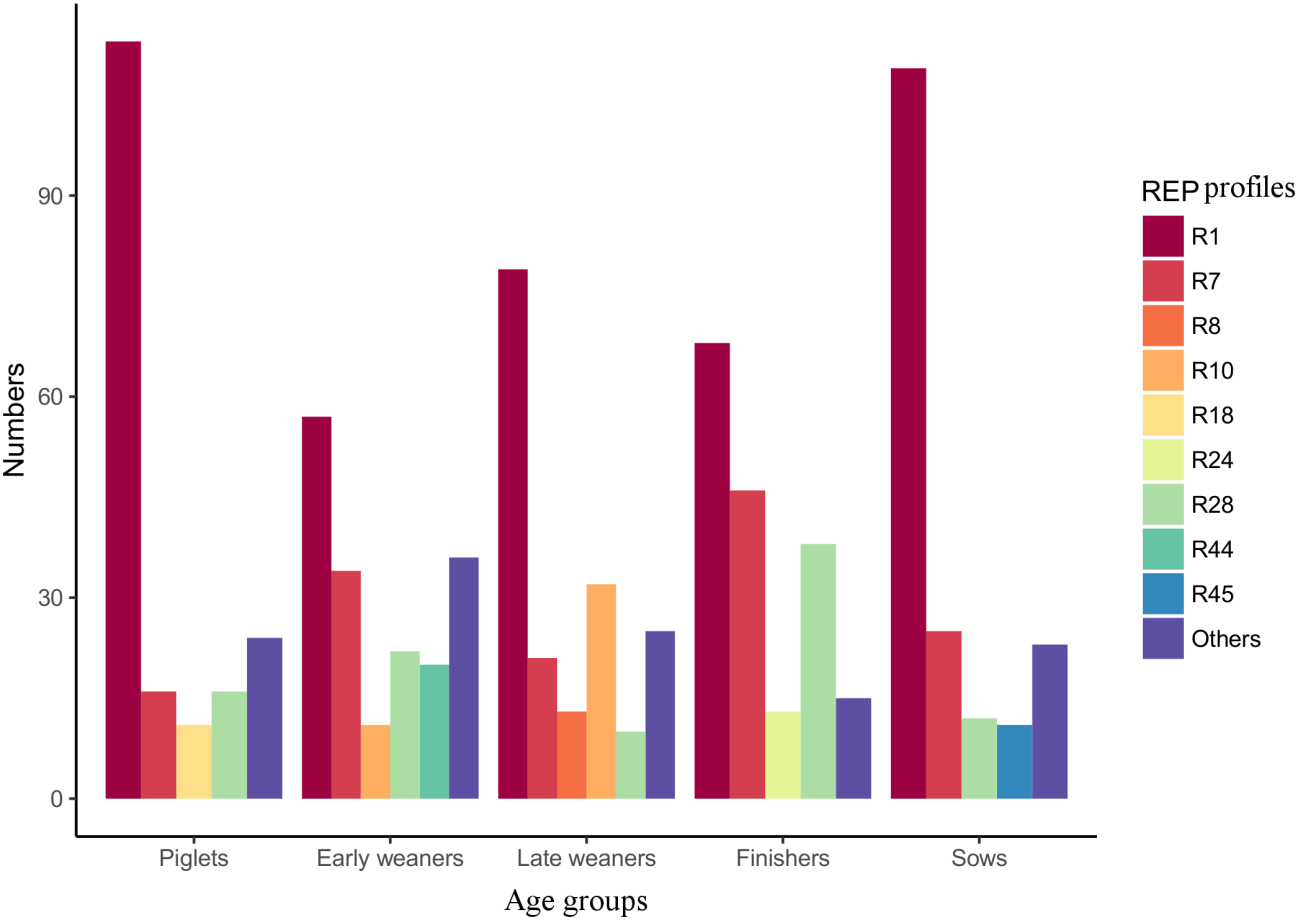
Distribution of REP profiles among the five age groups of pigs. Dominant REP profiles represented by at least 10 strains are shown. The sum corresponding to the rest of the REP profiles detected in each age group is termed as “Others”.

A total of 900 confirmed *E*. *coli* isolates were selected for the genetic diversity study. Here, we tested 45 *E*. *coli* isolates per fecal sample. In a previous study, we demonstrated that 10 colonies per pig should be enough to represent the genetic diversity of a single animal [19]. REP-PCR DNA fingerprint showed a high genetic diversity among the strains, both between the five age groups of pigs and within each of the groups, as well as at the pig level (Fig 1, S1 Table). A total of 52 unique REP profiles were identified from all the 900 *E*. *coli* strains tested. The most frequent REP profiles detected were R1 (47%, N = 426) followed by R7 (16%, N = 142) (S2 Table, S1 Fig). Among the age groups, the highest and lowest number of different REP profiles were found in early weaners (N = 24) and in finishers (N = 13) respectively (S2 Fig). The number of different REP profiles in each of the 20 pigs analyzed in the study ranged from 2 to 13 (S1 Table).

Shannon diversity index (*H'*) showed that the overall diversity index was 2.05. The highest diversity was observed in early weaner pigs, with an *H'* of 2.22 followed by late weaner pigs (*H'*, 1.91). The lowest index was detected in piglets (*H'*, 1.46), even though finishers had an overall lower number of types (Table 1). However, no statistical significance was detected between the numbers of different REP profiles obtained for each age group as determined by ANOVA analysis and Tukey's multiple comparison test (not shown).

**Table 1:** Shannon diversity index (*H'*) obtained for each age group of pigs.

Age groups	No. of isolates	No. of unique REP profiles	*H’*
Piglets	180	17	1.46
Early weaners	180	24	2.22
Late weaners	180	21	1.91
Finishers	180	13	1.61
Sows	180	17	1.49
Total	900	52	2.05

**Fig 2:**
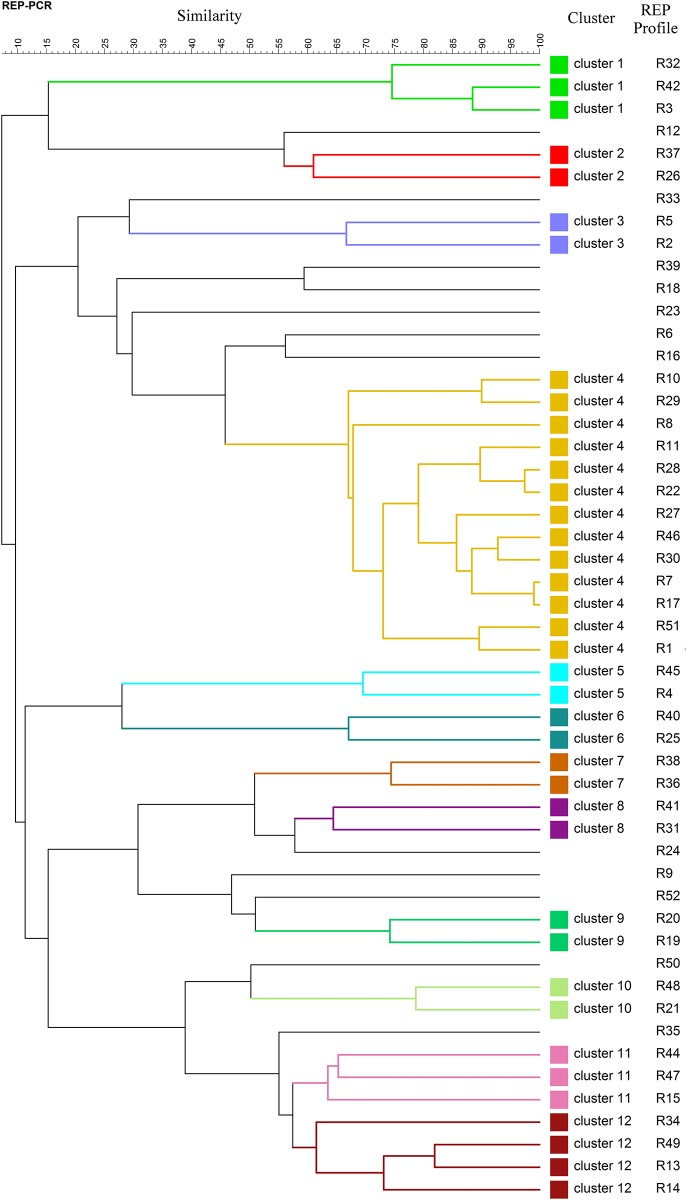
Dendrogram showing the relatedness of all REP-PCR fingerprints generated from *E*. *coli* strains isolated from all pigs included in the study. Pearson coefficient was used to calculate REP profiles similarities and the dendrogram was generated by UPGMA.

A dendrogram was generated from the curve based Pearson co-efficient of the 52 unique REP fingerprints observed, and this was used to assess the first rough estimate of phylogenetic relationship between profiles (Fig 2). The dendrogram showed 12 distinct clusters at 60% cut off value, each containing at least two strains, and with the three most common REP profiles (R1, R7 and R28) belonging to the same cluster.

**Fig 3:**
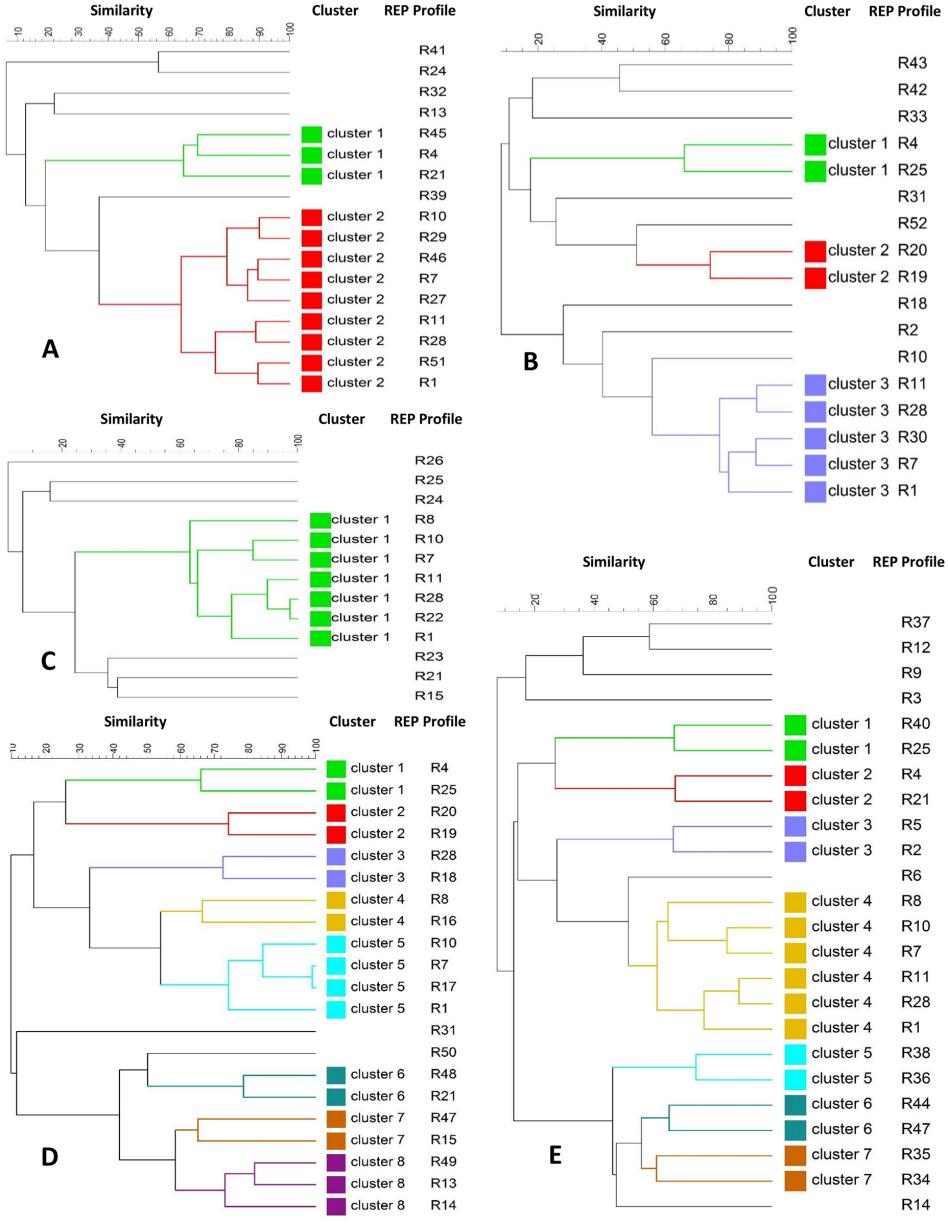
Dendrogram showing the relatedness of REP-PCR fingerprints of *E*. *coli* strains within each age group of pigs including sows (A), piglets (B), finishers (C), late weaners (D) and early weaners (E). Pearson coefficient was used to calculate REP profiles similarities and the dendrogram was generated by UPGMA.

To understand the genetic diversity of *E*. *coli* isolates within each of the age group of pigs, dendrograms were constructed for every single age group (Fig 3). Within the group of piglets (Fig 3B) the 17 unique REP profiles identified were grouped into three clusters (cluster 1 and 2 with 2 isolates in each and cluster 3 with 5 isolates) at a 60% similarity cut off value, and the rest of the isolates represented singleton lineages (similarity ranging from 18% to 55%). The dendrogram encompassing the 24 different *E*. *coli* isolates observed in the group of early weaners (Fig 3E) showed 7 small clusters comprising 2–6 isolates, and six singleton lineages. The 21 different isolates from late weaners formed eight clusters (N = 19 isolates), and only two unique singleton lineages (Fig 3D). In the group of finishers, there was only one big cluster encompassing seven isolates, and the rest of the isolates were singleton lineages (Fig 3C). Finally, in the group of isolates from sows, only two clusters were observed, one big cluster with nine isolates, and another one with three isolates (Fig 3A).

### PFGE typing of strains

**Fig 4:**
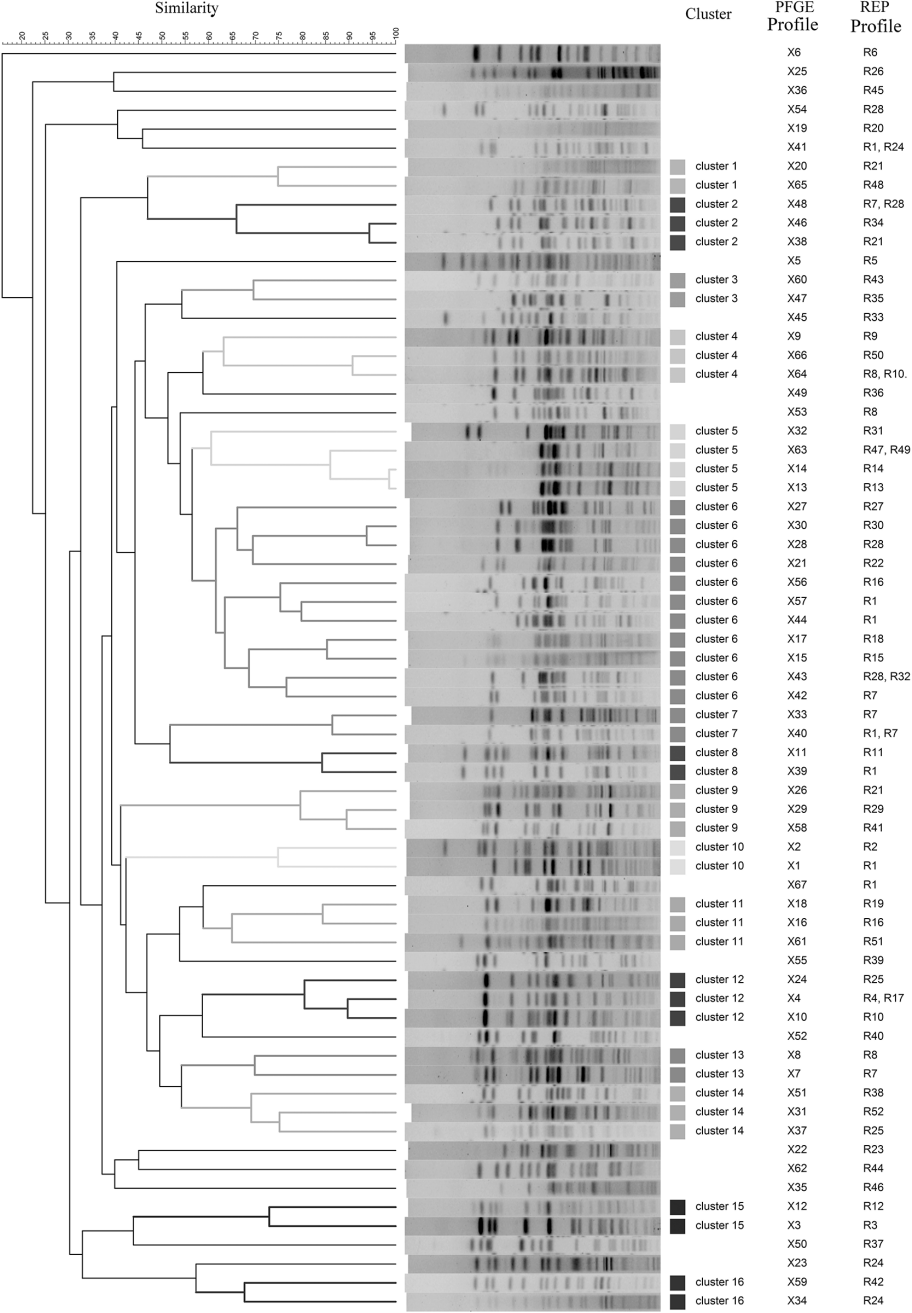
Pulsed field gel electrophoresis (PFGE) fingerprints and dendrogram showing the relatedness of PGFE types generated from *E*. *coli* strains. Clonal relatedness was calculated by using curve-based Pearson coefficient. REP profile numbers are shown next to the PFGE pattern numbers.

Next, PFGE was used to determine more precisely the genetic diversity of *E*. *coli* strains showing different REP-PCR profiles. In addition to one isolate assigned to each of the 52 unique REP profiles, 24 isolates associated with the most frequent REP profiles [R1 (N = 6), R7 (N = 4), R28 (N = 3), R8 (N = 2), R21 (N = 2), R24 (N = 2), R10 (N = 1), R16 (N = 1), R25 (N = 1), R45 (N = 1) and R47 (N = 1)] were also analyzed. Therefore, a total of 76 isolates were subjected to PFGE analysis (Fig 4) and 67 distinct PFGE patterns were obtained. Some of the different REP fingerprints shared common PFGE patterns; for example, PFGE pattern X41 encompassed REP profiles R1 and R24 and X48 included R7 and R28 (Fig 4), but in general strains shown to differ by REP analysis also differed by PFGE. On the other hand, some strains assigned to the same REP profile led to different PFGE patterns, i.e.: seven isolates assigned to R1 profile displayed seven different PFGE patterns (X1, X39, X40, X41, X44, X57 and X67) clearly showing that PFGE is more sensitive to show strain differences than REP-PCR, and only two isolates assigned to the same REP profile (R45) showed the same PFGE pattern (X36) (Fig 4, S3 Table). A dendrogram was constructed based on the PFGE patterns observed. Sixteen different clusters containing at least two unique PFGE patterns were identified, using a 60% similarity cut off value (Fig 4). Of these, cluster 6 contained the largest number of PFGE patterns (N = 11), with fingerprints similarity ranging from 60 to 94%. Eighteen PFGE patterns remained as singleton lineages with 10–58% fingerprint similarity. The most common REP profiles, R1, R7 and R28, for which several associated strains were analyzed, clustered together for the most of the strains analyzed, however, exceptions were observed (Fig 4).

### WGS analysis

**Fig 5:**
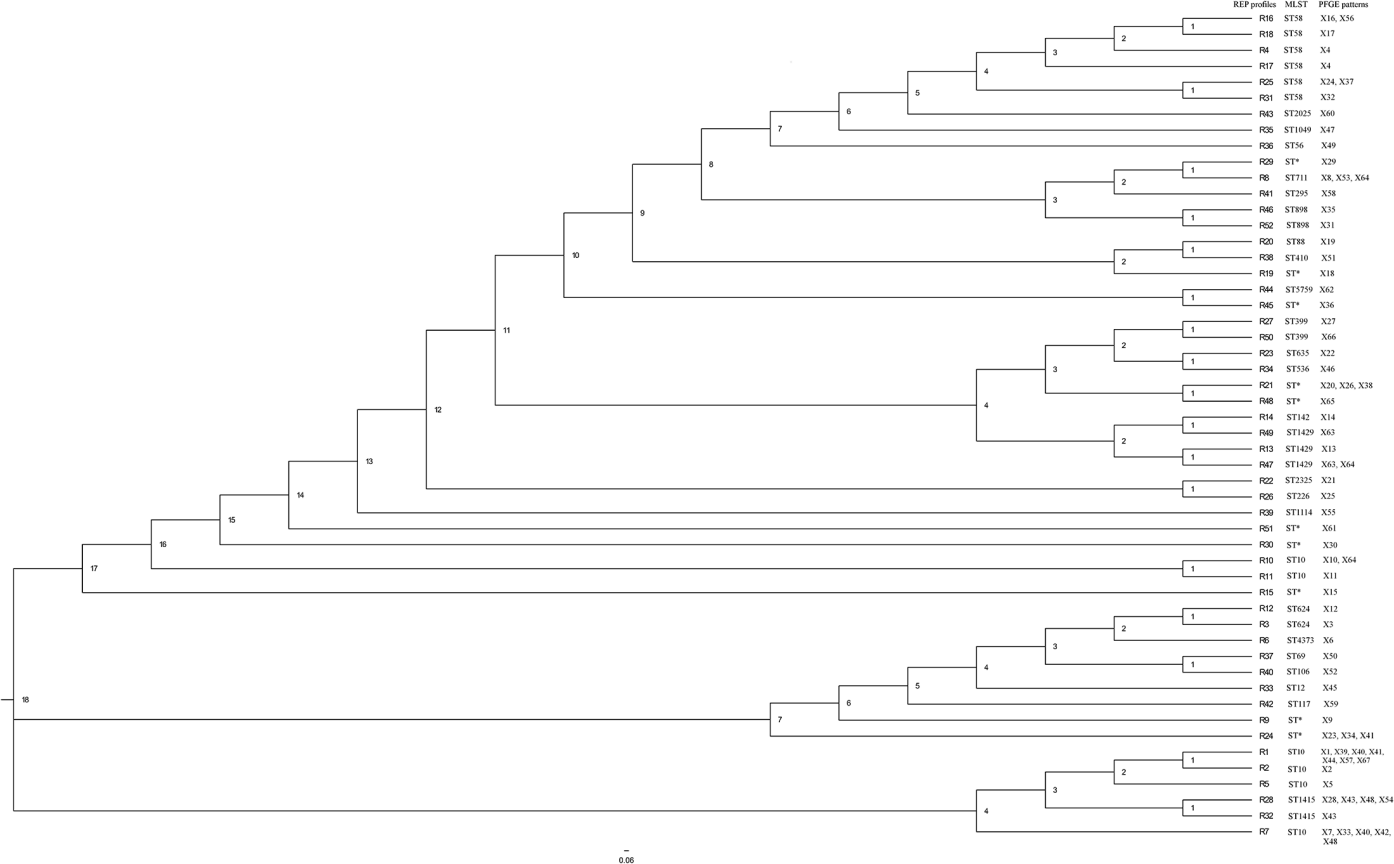
SNP-based phylogeny of *E*. *coli* representing the 52 unique REP profiles generated by the commensal strains isolated from the pigs under study. Phylogeny was inferred with SNP procedure by using the center for genomic epidemiology tool CSI phylogeny 1.4. Default filtering option was applied during SNP calling. Node labels are shown as decimal. ST*, unknown sequence type.

The fifty-two isolates assigned to unique REP profiles were further subjected to WGS. Paired end raw reads were submitted to ENA under the study accession number PRJEB15511, and the specific accession numbers for each of the 52 genomes are listed in Table 2. K-mer based species identification confirmed that all of them were *E*. *coli*. A total of 25 MLST types were identified for 42 isolates, while 10 isolates showed an unidentified MLST type. Seven MLST types were linked to several REP profiles, for example the most common sequence types, ST10 and ST58, as well as ST1429, ST399, ST624, ST898 and ST1415, which were associated with six, six, four, two, two and two different REP profiles, respectively (Table 2). Thirty seven serotypes were detected for the 52 isolates. Eight of them were associated with several REP profiles, being serotype O19:H7 the most frequently observed (associated with six different REP profiles). We could not confirm the O specific type of 11 isolates (Table 2). SNP analysis showed a wide genetic diversity among the isolates. The different number of SNPs ranged from 1 (between R10 and R11) to 39789 (R33 and R45) (S3 Fig). Interestingly, the three most common profiles, R1, R7 and R28 were grouped in the same cluster and the most distant profile was R33. A total of 3583, 3909 and 5055 different SNPs were observed between R1 and R7, R1 and R28, and R7 and R28, respectively. These data suggest that the most frequent commensal *E*. *coli* strains from pigs are closely related, which is supported by the fact that they all belonged to the same phylogenetic group (Fig 5). Many PFGE patterns from a same cluster in the PFGE dendrogram (Fig 4) are also closely related in SNP phylogeny (Fig 5). However, very closely related REP profiles from the same cluster in the REP dendrogram (Fig 2) are distantly related in the SNP phylogeny (Fig 5).

**Table 2:** Genetic features of commensal *E*. *coli* isolates belonging to different REP profiles based on whole genome sequence analysis.

Isolates	REP profiles	Resistance genes	Virulence genes	MLST type	Plasmids	Serotype	Accession Number
P1.08	R1	-	*astA*, *gad*, *iss*	ST10	-	O^-^:H11	ERS1363800
P1.20	R2	*mph*(A)-like	*astA*, *celb*, *iss*	ST10	IncFII, Col(BS512), Col8282, ColRNAI, Col156, Col(MG828)	O13:H11	ERS1363801
P1.49	R3	*sul*2	*air*, *gad*	ST624	ColRNAI	O^-^:H1	ERS1363802
P1.51	R4	*str*B	*iss*, *lpfA*	ST58	ColRNAI	O8:H10	ERS1363803
P1.53	R5	*bla*_TEM-1B_, *sul*2	*astA*, *gad*, *iss*	ST10	IncFII, ColRNAI	O^-^:H11	ERS1363804
P1.56	R6	*aad*A1, *bla*_TEM-1B_, *cat*A1-like, *dfr*A1, *str*A, *str*B, *sul*2, *tet*(B)	*air*, *astA*, *gad*, *iss*, *lpfA*	ST4373	IncQ1	O^-^:H34	ERS1363805
P1.57	R7	*aad*A1, *bla*_TEM-1C_-like, *mph*(A)	*iss*	ST10	IncFII, IncX1	O^-^:H19	ERS1363806
P1.69	R8	*bla*_TEM-1B_-like, *tet*(A)	*astA*, *celb*, *lpfA*	ST711	IncX1, Col(MG828), Col156, ColRNAI	O^-^:H25	ERS1363807
P1.75	R9	*aad*A1, *str*B	*gad*, *ireA*, *iroN*, *iss*, *lpfA*, *pic*	Unknown ST	IncFIB(AP001918), IncI1, IncFIC(FII), ColRNAI, Col(MG828)	O8:H4	ERS1363808
P1.77	R10	*-*	*gad*	ST10	IncHI2A, IncHI2, IncX1, ColRNAI	O109:H27	ERS1363809
P1.78	R11	*-*	*gad*	ST10	IncHI2A, IncFIA, IncHI2, IncX1, ColRNAI	O109:H27	ERS1363810
P1.85	R12	*sul*2	*air*, *eilA*, *gad*, *lpfA*	ST624	ColRNAI	O^-^:H1	ERS1363811
P1.99	R13	*-*	*celb*, *gad*, *lpfA*	ST1429	IncP(6), IncX1, Col156, ColRNAI	O19:H7	ERS1363812
P1.101	R14	*-*	*celb*, *gad*, *lpfA*	ST1429	IncX1, IncP(6), Col156, ColRNAI	O19:H7	ERS1363813
P1.115	R15	*aad*A1, *dfrA*5, *str*A, *str*B-like	*astA*, *ccI*, *iroN*, *lpfA*	Unknown ST	IncFIB(K), IncFII, IncFIB(AP001918), IncFII(pCRY), Col(MG828), ColRNAI	O9:H19	ERS1363814
P1.123	R16	*dfr*A5	*cba*, *gad*, *iss*, *lpfA*, *mchF*	ST58	IncFII, IncI1, IncFIB(AP001918), ColRNAI	O8:H10	ERS1363815
P1.124	R17	*bla*_TEM-1B_*-*like, *dfr*A5	*cba*, *cma*, *gad*, *iss*, *lpfA*, *mchF*	ST58	IncFII, IncI1, IncFIB(AP001918), ColRNAI	O8:H10	ERS1363816
P1.127	R18	*dfr*A5, *str*A, *sul*2	*cba*, *cma*, *gad*, *iss*, *lpfA*, *mchF*	ST58	IncFII, IncI1, IncFIB(AP001918), ColRNAI	O8:H10	ERS1363817
P1.134	R19	*bla*_TEM-1B_, *dfr*A5	*iroN*, *iss*, *lpfA*, *mchF*	Unknown ST	IncFIB(AP001918), IncX1	O21:H21	ERS1363818
P1.135	R20	*dfr*A5, *str*A, *str*B, *sul*2	*gad*, *iss*, *lpfA*, *mchF*	ST88	IncFIB(AP001918)	O8:H19	ERS1363819
P1.154	R21	*-*	*gad*, *lpfA*	Unknown ST	IncY	O19:H7	ERS1363820
P1.173	R22	*-*	*iss*	ST2325	-	O66:H25	ERS1363821
P1.176	R23	*-*	*gad*, *lpfA*	ST635	ColRNAI	O11:H25	ERS1363822
P1.187	R24	*-*	*ireA*, *iss*, *lpfA*, *mchB*, *mchC*, *mchF*	Unknown ST	IncFIC(FII), IncFIB(AP001918), ColRNAI	O127:H4	ERS1363823
P2.08	R25	*aph*(3')-Ia-like, *bla*_TEM-1B_, *dfr*A5, *str*B-like	*gad*, *iroN*, *iss*, *lpfA*	ST58	IncFII, IncFIB(AP001918), ColRNAI	O8:H19	ERS1363824
P1.192	R26	*-*	*gad*	ST216	IncFIA(HI1), IncHI1A, IncHI1B(R27), IncFIB(K), p0111	O3:H4	ERS1363825
P1.204	R27	*-*	*gad*, *lpfA*	ST399	IncY, ColRNAI	O13/O135:H3	ERS1363826
P2.200	R28	*-*	*astA*, *iss*	ST1415	IncFII	O108:H34	ERS1363827
P1.238	R29	*-*	*astA*, *gad*, *iss*, *lpfA*	Unknown ST	IncFIC(FII), IncFIB(AP001918), ColRNAI	O70:H10	ERS1363828
P2.02	R30	*-*	*astA*, *iss*, *lpfA*	Unknown ST	IncFIB(AP001918), ColRNAI	O60:H5	ERS1363829
P2.19	R31	*bla*_TEM-1B_, *dfr*A5,*strA*, *str*B-like, *sul*2-like	*iroN*, *iss*, *lpfA*, *mchF*	ST58	IncFII, IncFIB(AP001918), ColRNAI	O8:H19	ERS1363830
P2.194	R32	-	*astA*, *gad*, *iss*	ST1415	IncFII	O108:H34	ERS1363831
P3.01	R33	-	*cnf1*, *gad*, *iroN*, *iss*, *mchB*, *mchC*, *mchF*, *mcmA*, *vat*	ST12	ColRNAI	O4:H5	ERS1363832
P3.52	R34	-	*gad*, *lpfA*	ST536	IncY	O154:H9	ERS1363833
P3.62	R35	-	*gad*, *lpfA*	ST1049	ColRNAI	O32:H10	ERS1363834
P3.69	R36	*bla*_TEM-1B_, *tet*(A)	*iroN*, *iss*, *lpfA*, *mchF*	ST56	IncFIB(AP001918), IncX1	O21:H21	ERS1363835
P3.71	R37	*bla*_TEM-1B_	*air*, *eilA*, *gad*, *iss*, *lpfA*, *mchB*, *mchC*, *mchF*, *mcmA*, *tsh*	ST69	IncFIC(FII), IncFII(29), IncFIB(AP001918)	O15:H6	ERS1363836
P3.75	R38	-	*iss*, *lpfA*	ST410	IncFIB(AP001918), Col(MG828), ColRNAI	O^-^:H9	ERS1363837
P3.203	R39	-	*gad*	ST1114	Col8282, ColRNAI	O98:H26	ERS1363838
P3.96	R40	-	*air*, *eilA*, *gad*, *iss*, *lpfA*	ST106	IncFII	O17/O44:H18	ERS1363839
P3.235	R41	-	*astA*, *gad*, *lpfA*	ST295	IncFII, IncFIB(AP001918)	O171:H21	ERS1363840
P4.25	R42	-	*gad*, *ireA*, *iss*, *lpfA*, *mchB*, *mchC*, *mchF*, *vat*	ST117	ColRNAI	O114:H4	ERS1363841
P4.31	R43	-	*gad*, *iroN*, *iss*, *lpfA*	ST2025	ColRNAI	O8:H25	ERS1363842
P4.53	R44	-	*air*, *astA*, *eilA*, *iss*, *stb*	ST5759	IncFII(29), IncFIB(AP001918)	O^-^:H20	ERS1363843
P4.194	R45	-	*gad*	Unknown ST	Col8282, Col156, ColpVC, Col(MG828)	O^-^:H7	ERS1363844
P4.200	R46	*aad*A1, *tet*(B)	*lpfA*	ST898	Col(MG828)	O154:H48	ERS1363845
P4.88	R47	-	*celb*, *gad*	ST1429	IncP(6), IncX1, Col156, ColRNAI	O19:H7	ERS1363846
P4.132	R48	-	*gad*, *lpfA*	Unknown ST	Col(MGD2), ColRNAI, IncY, IncX1, IncL/M(pOXA-48), Col(Ye4449)	O19:H7	ERS1363847
P4.136	R49	-	*celb*, *lpfA*	ST1429	IncX1, IncP(6), Col156, ColRNAI	O19:H7	ERS1363848
P4.143	R50	-	*gad*, *lpfA*	ST399	IncY	O13/O135:H30	ERS1363849
P4.233	R51	-	*-*	Unknown ST	IncFII(29), Col8282, ColE10, Col156, ColpVC, Col(MG828)	O142:H38	ERS1363850
P2.09	R52	*aad*A1, *bla*_TEM-1B_-like, *dfr*A1	*iss*, *lpfA*	ST898	IncY, ColRNAI	O^-^:H48	ERS1363851

-, not identified; resistance phenotype- Aminoglycoside (*aad*A1, *aph*(3')-Ia-like, *str*A, *str*B-like), Trimethoprim (*dfr*A1, *dfr*A5), Beta lactam (*bla*_TEM-1B,_ *bla*_TEM-1B_-like), Tetracycline (*tet*A, *tet*B), Sulphonamide (*sul*2, *sul*2-like), Macrolide (*mph*A, *mph*A-like), Phenicol (*cat*A1-like); Virulence- Enteroaggregative immunoglobulin repeat protein (*air*), Heat-stable enterotoxin 1 (*astA*), colicin B (*cba*), cloacin (*ccI*), endonuclease colicin E2 (*celb*), colicin M (*cma*), Cytotoxic necrotizing factor (*cnf1*), Salmonella HilA homolog (*eilA*), Glutamate decarboxylase (*gad*), Siderophore receptor (*ireA*), enterobactin siderophore receptor protein (*iroN*), increased serum survival (*iss*), long polar fimbriae (*lpfA*), microcin H47 part of colicin H (*mchB*), MchC protein (*mchC*), ABC transporter protein MchF (*mchF*), microcin M part of colicin H (*mcmA*), serine protease autotransporters of *Enterobacteriaceae* (*pic*), heat-stabile enterotoxin II (*stb*), serine protease autotransporters of *Enterobacteriaceae* (*tsh*), serine protease autotransporters of *Enterobacteriaceae* (*vat*)

Only 21 out of the 52 sequenced commensal *E*. *coli* stains were shown to carry DNA with significant homology to known antimicrobial resistance genes. A total of 14 different antimicrobial resistance genes were identified in these 21 isolates conferring resistance to seven classes of antimicrobials. The strain assigned to the most common REP profile, R1 was included among the thirty-one strains that did not harbor any of the antimicrobial resistance genes analyzed. Also, remarkably, none of the strains contained known ESBL (Extended Spectrum Beta-Lactamase) genes. Eleven strains harbored resistance genes either for aminoglycoside and/or beta-lactam antimicrobials. Fifteen isolates had more than one resistance gene (Table 2). We identified 29 different plasmid replicons, and 50 isolates out of 52 analyzed were shown to carry such signature sequences of plasmids and other mobile genetic elements. The most abundant replicon was ColRNAI, which was detected in 32 isolates followed by IncFIB in 17 isolates. Thirty-four isolates harbored multiple plasmid replicons.

At least one virulence gene was detected in 51 isolates out of the 52 sent for sequencing. Twenty-one different virulence genes were identified, of which the most frequent ones were *lpfA* (long polar fimbriae), *gad* (glutamate decarboxylase) and *iss* (increased serum survival) identified in 35, 34 and 29 isolates, respectively (Table 2).

## Discussion

In a previous study, we analyzed the genetic diversity of *E*. *coli* in the gut of nursery pigs from several Danish farms, where antibiotic treatment was commonly administered at one or more time points during the production cycle [2]. So far, the genetic diversity and relatedness of commensal *E*. *coli* in the gut of non-antimicrobial treated pigs has never been analyzed. Moreover, we do not know how the diversity varies between different age groups.

It is known that the genetic structure of the intestinal *E*. *coli* population is determined by multiple host and environmental factors [1]. In our previous study, we observed a larger number of different *E*. *coli* strains in a farm where the particular group of pigs analyzed were accidently not treated with antimicrobials (only zinc oxide was administered) compared to the rest of groups including treated pigs from other farms. Thus, we suggested that the higher diversity detected could be due to the lack of antimicrobial treatment of the animals during the nursery period [2]. In the current study, we observed a high degree of diversity within and between the different age groups of pigs in the absence of antimicrobial treatment. The highest diversity was observed within the group of early weaners (this was also the age of the pigs from the groups tested in our previous studies). However, the diversity was not remarkably higher than diversities observed in groups of nursery pigs from production systems where use of antimicrobials is common [2,19], suggesting that antimicrobial treatment may not be as important in determining genetic diversity of the *E*. *coli* commensal microbiota as previously assumed. Nevertheless, a precise comparison of the intestinal *E*. *coli* genetic diversity between groups of pigs that originate in the same herd and which just undergo different treatments (treated with different antimicrobials and not treated) might be carried out in order to study this further. A possible confounding factor in our observation is the fact that antibiotic free production systems have been recently implemented. This means that the piglets are still born from sows that have been raised in environments where use of antimicrobials is common, and this may affect the diversity of their microbiota.

The most common profile in the current study (R1) was also the most commonly observed in a previous study on diversity of commensal *E*. *coli* from pigs [2]. In that study, strains assigned to unique REP profiles were characterized by DNA microarray, supplying most of the information obtained by WGS in the current study. An important observation is, that the strain assigned to R1 which was sequenced in the current study did not carry any resistance genes, while the R1 strain in the previous study [2] was shown to carry aminoglycoside, β-lactam and tetracycline resistance genes, suggesting that the effect of the antimicrobial treatment may be the acquisition and maintenance of resistance genes in well adapted commensal *E*. *coli* strains. Further studies into this interesting observation are warranted.

As previously demonstrated [2,19], our results indicated that a single pig generally harbors one predominant strain of *E*. *coli* strain together with one to few other strains. However, overall, REP-PCR DNA fingerprint analysis showed a substantial genetic diversity between *E*. *coli* strains regardless of the age of the pigs. Wide ranges of genetic diversity were also demonstrated by others in studies on diversity of commensal *E*. *coli* from humans and from other animals [15,27–29].

We observed that genetic diversity in commensal *E*. *coli*, although no significant differences were detected, varied depending on the age group with *E*. *coli* from early weaner pigs showing the highest diversity. As suggested in previous studies [30,31] these findings indicate that age could be a contributing factor to the genetic diversity observed among commensal *E*. *coli*, even though the mechanisms are far from understood. The higher genetic diversity observed in early weaner pigs (not significant) compared to other age groups might be due to the physiology of pigs at this particular age. At the beginning of the weaning period, pigs undergo a stressful condition due to their separation from the mothers and transition of diet from milk based to a solid one [32] and this may disturb the microbiota so much that a high number of commensal *E*. *coli* strains supplied through the feed can establish themselves in the intestine. As the balance is re-established, only the adapted strains may be able to persist. It is possible that the low diversity detected in piglets is a reflection of the rather low diversity in the intestine of the sows, since the piglets are believed to mainly get their strains directly from the mother [3]. Other host and environmental factors, apart from the age, and diet, might be important, and it would be interesting to study how herd specific factors can influence the diversity of the commensal *E*. *coli*, once a larger and stable antimicrobial free production system has been established.

As indicated in previous studies, [2,19], certain profiles (termed R1, R7 and R28 in the current study) were dominant. We speculate that strains assigned to these profiles represent the archetypal commensal *E*. *coli*. The three strains (associated with R1, R7 and R28) sequenced in the current study belonged to the sequence type ST10 (R1 and R7), and ST1415 (R28). The only strain for which we could determine the full serotype belonged to O108:H34 (R28). It would be of relevance to sequence more strains yielding these dominant profiles in the future in order to identify common factors that differentiate them from the less frequent strains.

The Danish pig production system is not strictly pyramidal, and therefore such widespread observed for the same type of commensal *E*. *coli* supports our speculation that they are particularly adapted for persistence in pigs under the current production conditions. Not only the ability to survive and grow in the intestine, but also the ability to spread between pigs must be important in this context. Besides, it has been shown that a strong selection of *E*. *coli* takes place following excretion into the environment which may explain why certain *E*. *coli* types could form stable populations [16]. Other parameters such as a common diet may also justify the occurrence of dominant clones [16,33].

Apart from REP-PCR (900 isolates were typed) we performed PFGE typing in order to characterize a sub-set of strains and it was demonstrated that a few patterns were more common than others. The fact that different REP profiles led to the same PFGE pattern and the other way around demonstrated that there is not a correlation between REP profiles and PFGE patterns and highlights the relevance of choosing the appropriate technique when analyzing genetic diversity, as previously observed [19,34]. Since we also obtained a SNP based phylogenetic tree, our study allowed to evaluate which of the typing methods that reflected overall DNA-similarity was the best. The results shown in Fig 5 demonstrated that PFGE typing had a better discriminatory power since, in most of the cases, similar PFGE patterns (Fig 4) clustered together in the SNP tree too. Interestingly, also, most of the same ST types clustered together in the SNP tree as it was shown for the six isolates assigned to ST58.

As previously demonstrated in a study of Danish pigs [2] none of the isolates under study harbored true ESBL genes, which can be explained by the fact that 3^rd^ and 4^th^ generation cephalosporins are not used in the pig production in Denmark [35]. However, contrary to our previous study, where the prevalence of one-antimicrobial resistance gene carriage was above 88% and two-thirds of the strains carried three or more resistance genes, the same rates in the current study were of 40.4% and 15.4%, respectively. The most abundant antimicrobial resistance genes found in the *E*. *coli* isolates in this study have also been detected in surveillance of *E*. *coli* from pigs in Denmark (DANMAP 2012, http://www.danmap.org), USA and France [34,36,37].

The 52 commensal isolates tested harbored a broad range of virulence genes. Seven strains contained at least one adhesion factor and one toxin gene, suggesting that 13% of randomly collected commensal *E*. *coli* could be potentially pathogenic strains, despite the samples were collected from healthy animals. Three of these seven isolates harbored multiple antimicrobial resistance genes, and six of them carried the gene for long polar fimbriae (*lpfA)* virulence gene that it has been described to be a potential virulence marker for pathogenic *E*. *coli* [38].

The dominant ST types were ST10 (six strains out of 52) and ST58 (six strains) which contrary to our observation, are most often reported as ESBL producing *E*. *coli* [39]. These two ST types are also commonly found in chickens, other animals and humans [39,40]. *E*. *coli* ST10 was also a dominant type in our previous study [2]. Among the ST types detected, we also have one strain assigned to ST117 which is a well-recognized avian pathogenic *E*. *coli* with zoonotic potential [41].

The dominant serotypes were O19:H7 (six isolates) followed by O8:H19 (four isolates) and O8:H10 (four isolates). However, the most frequent O antigen was O8 (nine strains) which is among one of the most commonly reported antigens associated with porcine pathogenic *E*. *coli* strains [42]. Other O antigens, such as O108 (two strains), usually linked to porcine pathogenic isolates [42] were also detected.

This and previous studies on genetic diversity might contribute to better characterize the commensal niche and to increase knowledge on the population genetics and their spread. They may also allow implementing accurate modeling studies with different purposes, such as modeling studies on emergence and selection of antimicrobial resistance.

## Supporting information

S1 Fig Frequency and distribution of different *E*. *coli* strains (REP profiles) within each age group of pigs. (TIFF)

S2 Fig Number of different *E*. *coli* strains (REP profiles) detected within each age group of pigs. (TIFF)

S3 Fig Distance matrix plot showing the number of SNPs difference between *E*. *coli* strains (REP profiles). (TIFF)

S1 Table REP profiles obtained for each single pig in the five age groups. (DOCX)

S2 Table Distribution of the REP profiles generated by the *E*. *coli* strains under study among the five age groups of pigs. (DOCX)

S3 Table PFGE patterns generated by the *E*. *coli* strains assigned to different REP profiles. (DOCX)
