# Supplementary figures and images for: The genetic diversity of commensal Escherichia coli strains isolated from non-antimicrobial treated pigs varies according to age group
Source: PLoS One. 2017 May 30;12(5):e0178623. doi: 10.1371/journal.pone.0178623 (PMC5448805; doi:10.1371/journal.pone.0178623)

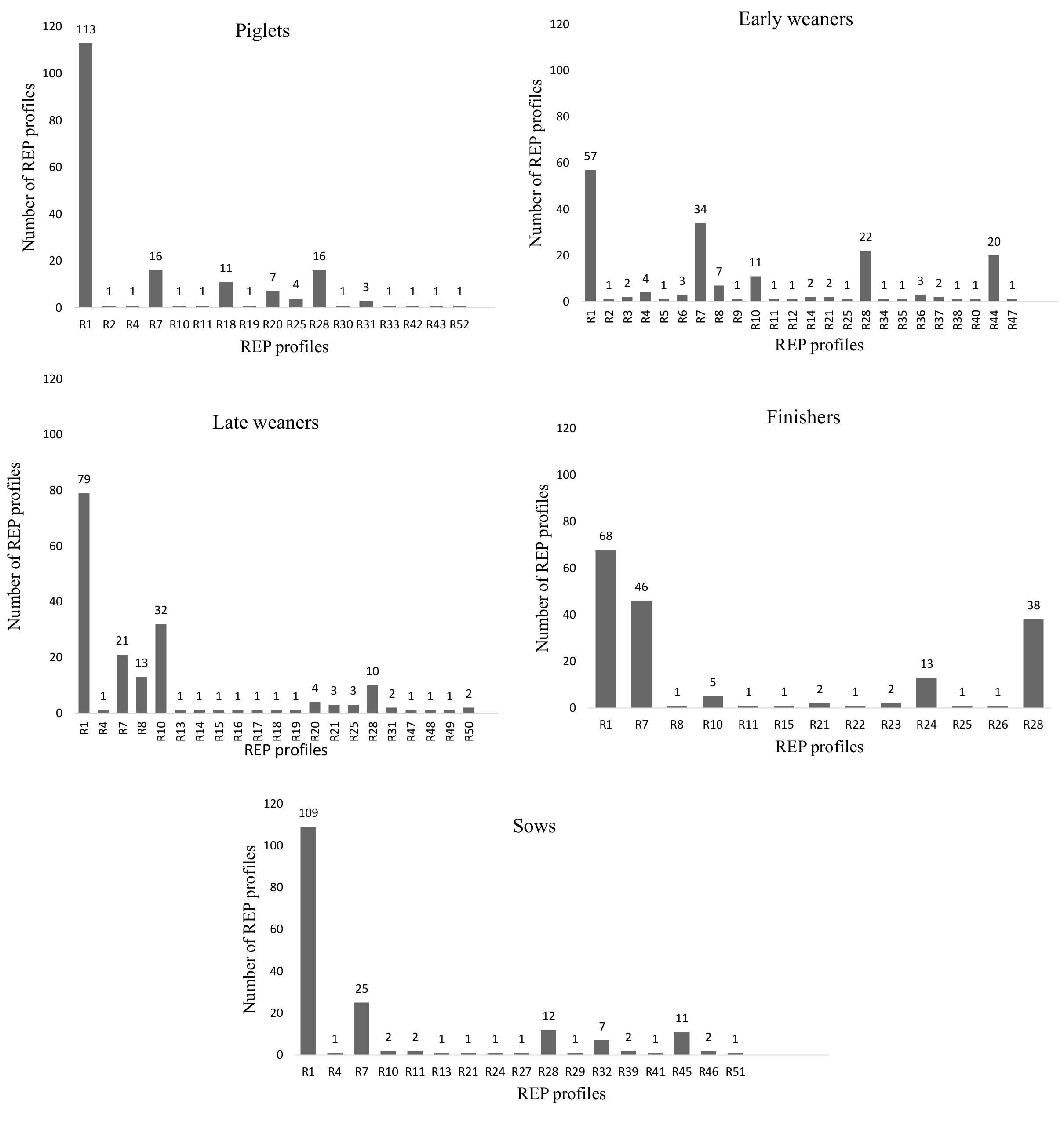

Supplement: S1 Fig — (TIFF) [file pone.0178623.s001.tiff]

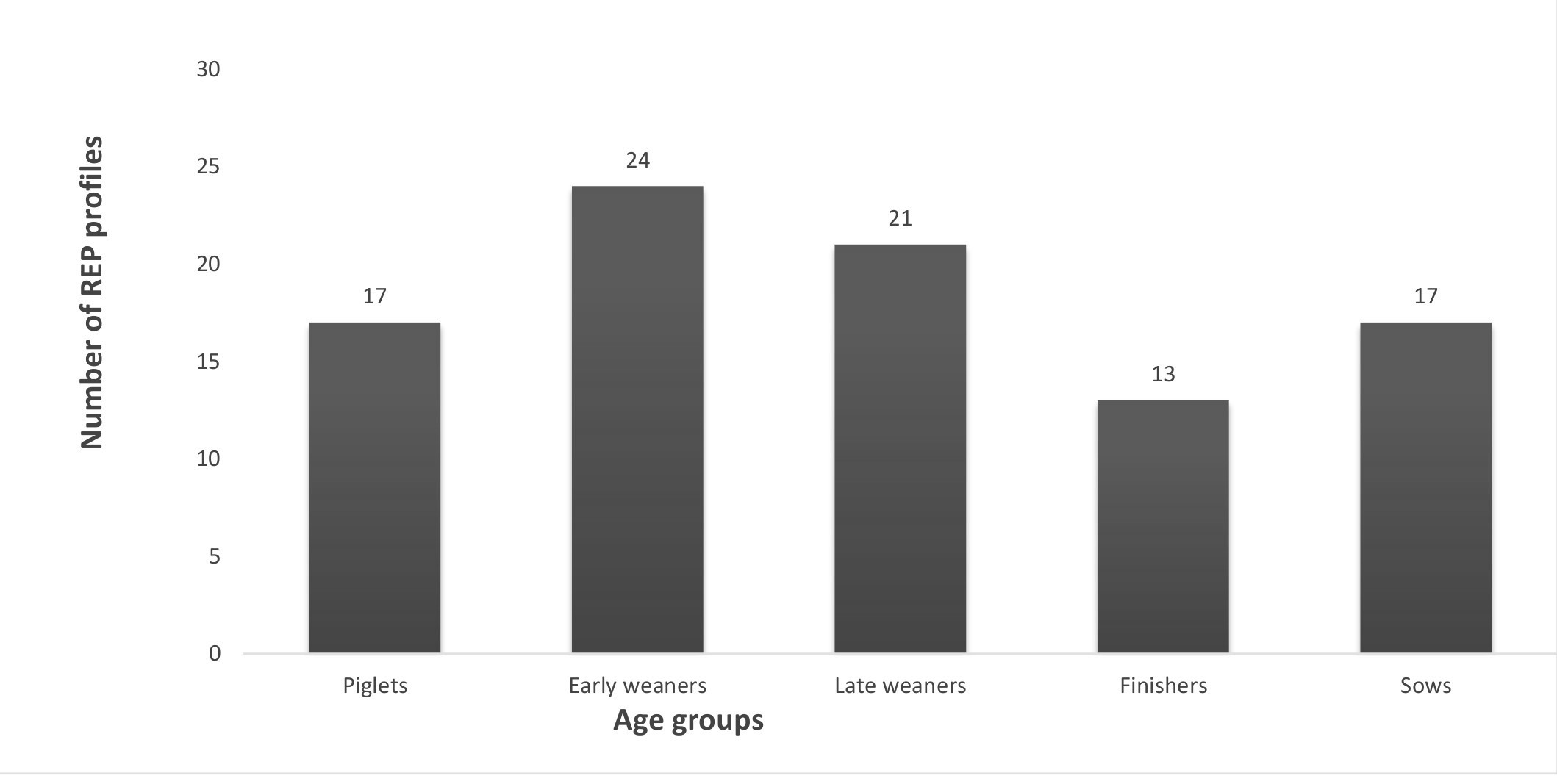

Supplement: S2 Fig — (TIFF) [file pone.0178623.s002.tiff]

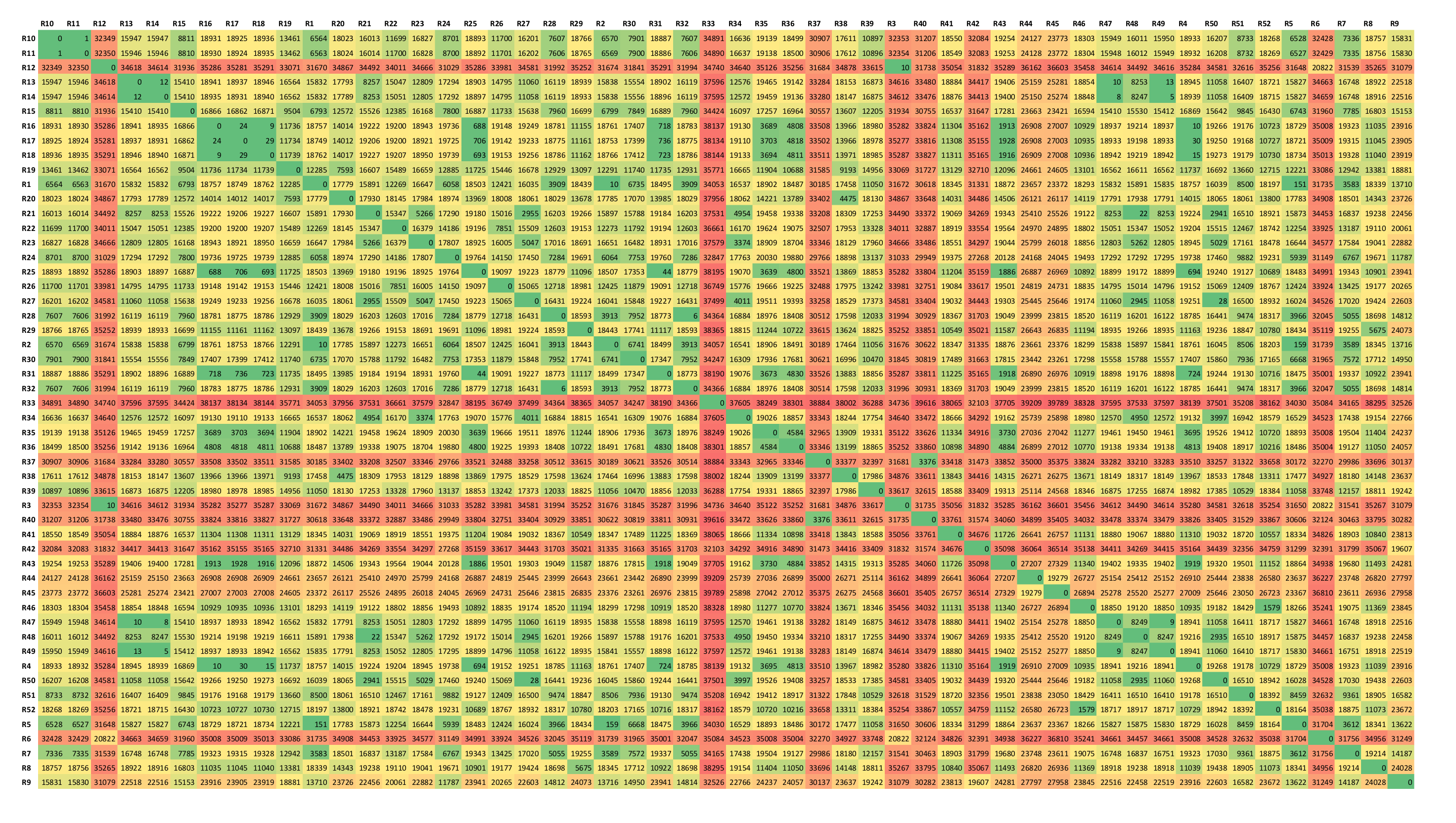

Supplement: S3 Fig — (TIFF) [file pone.0178623.s003.tiff]
