# Supplementary material for: The genetic diversity of commensal Escherichia coli strains isolated from non-antimicrobial treated pigs varies according to age group
Source: PLoS One. 2017 May 30;12(5):e0178623. doi: 10.1371/journal.pone.0178623 (PMC5448805; doi:10.1371/journal.pone.0178623)
Supplement: S1 Table — (DOCX) [file pone.0178623.s004.docx]

**Table S1**. REP profiles obtained for each single pig in the five age groups.

| **Age groups** | **Pigs** | **No. of profiles** | **REP profiles (N)** |
| --- | --- | --- | --- |
| Piglets | P1 | 2 | R1 (44), R2 (1) |
|  | P2 | 10 | R1 (24), R4 (1), R7 (4), R10 (1), R11 (1), R18 (7), R25 (2), R30 (1), R31 (3), R52 (1) |
|  | P3 | 8 | R1 (17), R7 (10), R18 (4), R19 (1), R20 (7), R25 (1), R28 (4), R33 (1) |
|  | P4 | 6 | R1 (28), R7 (2), R25 (1), R28 (12), R42 (1), R43 (1) |
| Early weaners | P1 | 12 | R1 (22), R2 (1), R3 (2), R4 (3), R5 (1), R6 (3), R7 (6), R8 (1), R9 (1), R10 (3), R11 (1), R12 (1) |
|  | P2 | 4 | R1 (25), R7 (13), R10 (5), R21 (2) |
|  | P3 | 13 | R1 (2), R4 (1), R7 (7), R8 (6), R14 (2), R25 (1), R28 (17), R34 (1), R35 (1), R36 (3), R37 (2), R38 (1), R40 (1) |
|  | P4 | 6 | R1 (8), R7 (8) , R10 (3), R28 (5), R44 (20), R47 (1) |
| Late weaners | P1 | 13 | R1 (21), R4 (1), R7 (7), R10 (4), R13 (1), R14 (1), R15 (1), R16 (1), R17 (1), R18 (1), R19 (1), R20 (4), R25 (1) |
|  | P2 | 4 | R1 (31), R7 (9), R21 (3), R31 (2) |
|  | P3 | 6 | R1 (25), R7 (5), R8 (9), R10 (2), R25 (1), R28 (3) |
|  | P4 | 9 | R1 (2), R8 (4), R10 (26), R25 (1), R28 (7), R47 (1), R48 (1), R49 (1), R50 (2) |
| Finishers | P1 | 9 | R1 (24), R7 (12), R11 (1), R21 (2), R22 (1), R23 (2), R24 (1), R25 (1), R26 (1) |
|  | P2 | 3 | R1 (16), R7 (17), R24 (12) |
|  | P3 | 4 | R1 (10), R7 (3), R10 (2), R28 (30) |
|  | P4 | 6 | R1 (18), R7 (14), R8 (1), R10 (3), R15 (1), R28 (8) |
| Sows | P1 | 8 | R1 (35), R4 (1), R7 (3), R11 (2), R21 (1), R27 (1), R28 (1), R29 (1) |
|  | P2 | 4 | R1 (14), R7 (18), R28 (6), R32 (7) |
|  | P3 | 8 | R1 (32), R7 (3), R10 (2), R13 (1), R28 (3), R39 (2), R41 (1), R46 (1) |
|  | P4 | 7 | R1 (28), R7 (1), R24 (1), R28 (2), R45 (11), R46 (1), R51 (1) |

N= number of isolates
