# Supplementary material for: The genetic diversity of commensal Escherichia coli strains isolated from non-antimicrobial treated pigs varies according to age group
Source: PLoS One. 2017 May 30;12(5):e0178623. doi: 10.1371/journal.pone.0178623 (PMC5448805; doi:10.1371/journal.pone.0178623)
Supplement: S2 Table — (DOCX) [file pone.0178623.s005.docx]

**Table S2.** Distribution of the REP profiles generated by the *E. coli* strains under study among the five age groups of pigs.

| REP profiles | Age groups, %REP profiles (No. of REP profiles) | | | | | |
| --- | --- | --- | --- | --- | --- | --- |
|  | *Piglets* | *Early weaners* | *Late weaners* | *Finishers* | *Sows* | *Total* |
| R1 | 62.8 (113) | 31.7 (57) | 43.9 (79) | 37.8(68) | 60.6 (109) | 47.3 (426) |
| R2 | 0.6 (1) | 0.6 (1) | 0.0 (0) | 0.0 (0) | 0.0 (0) | 0.2 (2) |
| R3 | 0.0 (0) | 1.1 (2) | 0.0 (0) | 0.0 (0) | 0.0 (0) | 0.2 (2) |
| R4 | 0.6 (1) | 2.2 (4) | 0.6 (1) | 0.0 (0) | 0.6 (1) | 0.8 (7) |
| R5 | 0.0 (0) | 0.6 (1) | 0.0 (0) | 0.0 (0) | 0.0 (0) | 0.1 (1) |
| R6 | 0.0 (0) | 1.7 (3) | 0.0 (0) | 0.0 (0) | 0.0 (0) | 0.3 (3) |
| R7 | 8.9 (16) | 18.9 (34) | 11.7 (21) | 25.6 (46) | 13.9 (25) | 15.8 (142) |
| R8 | 0.0 (0) | 3.9 (7) | 7.2 (13) | 0.6 (1) | 0.0 (0) | 2.3 (21) |
| R9 | 0.0 (0) | 0.6 (1) | 0.0 (0) | 0.0 (0) | 0.0 (0) | 0.1 (1) |
| R10 | 0.6 (1) | 6.1 (11) | 17.8 (32) | 2.8 (5) | 1.1 (2) | 5.7 (51) |
| R11 | 0.6 (1) | 0.6 (1) | 0.0 (0) | 0.6 (1) | 1.1 (2) | 0.6 (5) |
| R12 | 0.0 (0) | 0.6 (1) | 0.0 (0) | 0.0 (0) | 0.0 (0) | 0.1 (1) |
| R13 | 0.0 (0) | 0.0 (0) | 0.6 (1) | 0.0 (0) | 0.6 (1) | 0.2 (2) |
| R14 | 0.0 (0) | 1.1 (2) | 0.6 (1) | 0.0 (0) | 0.0 (0) | 0.3 (3) |
| R15 | 0.0 (0) | 0.0 (0) | 0.6 (1) | 0.6 (1) | 0.0 (0) | 0.2 (2) |
| R16 | 0.0 (0) | 0.0 (0) | 0.6 (1) | 0.0 (0) | 0.0 (0) | 0.1 (1) |
| R17 | 0.0 (0) | 0.0 (0) | 0.6 (1) | 0.0 (0) | 0.0 (0) | 0.1 (1) |
| R18 | 6.1 (11) | 0.0 (0) | 0.6 (1) | 0.0 (0) | 0.0 (0) | 1.3 (12) |
| R19 | 0.6 (1) | 0.0 (0) | 0.6 (1) | 0.0 (0) | 0.0 (0) | 0.2 (2) |
| R20 | 3.9 (7) | 0.0 (0) | 2.2 (4) | 0.0 (0) | 0.0 (0) | 1.2 (11) |
| R21 | 0.0 (0) | 1.1 (2) | 1.7 (3) | 1.1 (2) | 0.6 (1) | 0.9 (8) |
| R22 | 0.0 (0) | 0.0 (0) | 0.0 (0) | 0.6 (1) | 0.0 (0) | 0.1 (1) |
| R23 | 0.0 (0) | 0.0 (0) | 0.0 (0) | 1.1 (2) | 0.0 (0) | 0.2 (2) |
| R24 | 0.0 (0) | 0.0 (0) | 0.0 (0) | 7.2 (3) | 0.6 (1) | 1.6 (14) |
| R25 | 2.2 (4) | 0.6 (1) | 1.7 (3) | 0.6 (1) | 0.0 (0) | 1.0 (9) |
| R26 | 0.0 (0) | 0.0 (0) | 0.0 (0) | 0.6 (1) | 0.0 (0) | 0.1 (1) |
| R27 | 0.0 (0) | 0.0 (0) | 0.0 (0) | 0.0 (0) | 0.6 (1) | 0.1 (1) |
| R28 | 8.9 (16) | 12.2 (22) | 5.6 (10) | 21.1 (38) | 6.7 (12) | 10.9 (98) |
| R29 | 0.0 (0) | 0.0 (0) | 0.0 (0) | 0.0 (0) | 0.6 (1) | 0.1 (1) |
| R30 | 0.6 (1) | 0.0 (0) | 0.0 (0) | 0.0 (0) | 0.0 (0) | 0.1 (1) |
| R31 | 1.7 (3) | 0.0 (0) | 1.1 (2) | 0.0 (0) | 0.0 (0) | 0.6 (5) |
| R32 | 0.0 (0) | 0.0 (0) | 0.0 (0) | 0.0 (0) | 3.9 (7) | 0.8 (7) |
| R33 | 0.6 (1) | 0.0 (0) | 0.0 (0) | 0.0 (0) | 0.0 (0) | 0.1 (1) |
| R34 | 0.0 (0) | 0.6 (1) | 0.0 (0) | 0.0 (0) | 0.0 (0) | 0.1 (1) |
| R35 | 0.0 (0) | 0.6 (1) | 0.0 (0) | 0.0 (0) | 0.0 (0) | 0.1 (1) |
| R36 | 0.0 (0) | 1.7 (3) | 0.0 (0) | 0.0 (0) | 0.0 (0) | 0.3 (3) |
| R37 | 0.0 (0) | 1.1 (2) | 0.0 (0) | 0.0 (0) | 0.0 (0) | 0.2 (2) |
| R38 | 0.0 (0) | 0.6 (1) | 0.0 (0) | 0.0 (0) | 0.0 (0) | 0.1 (1) |
| R39 | 0.0 (0) | 0.0 (0) | 0.0 (0) | 0.0 (0) | 1.1 (2) | 0.2 (2) |
| R40 | 0.0 (0) | 0.6 (1) | 0.0 (0) | 0.0 (0) | 0.0 (0) | 0.1 (1) |
| R41 | 0.0 (0) | 0.0 (0) | 0.0 (0) | 0.0 (0) | 0.6 (1) | 0.1 (1) |
| R42 | 0.6 (1) | 0.0 (0) | 0.0 (0) | 0.0 (0) | 0.0 (0) | 0.1 (1) |
| R43 | 0.6 (1) | 0.0 (0) | 0.0 (0) | 0.0 (0) | 0.0 (0) | 0.1 (1) |
| R44 | 0.0 (0) | 11.1 (20) | 0.0 (0) | 0.0 (0) | 0.0 (0) | 2.2 (20) |
| R45 | 0.0 (0) | 0.0 (0) | 0.0 (0) | 0.0 (0) | 6.1 (11) | 1.2 (11) |
| R46 | 0.0 (0) | 0.0 (0) | 0.0 (0) | 0.0 (0) | 1.1 (2) | 0.2 (2) |
| R47 | 0.0 (0) | 0.6 (1) | 0.6 (1) | 0.0 (0) | 0.0 (0) | 0.2 (2) |
| R48 | 0.0 (0) | 0.0 (0) | 0.6 (1) | 0.0 (0) | 0.0 (0) | 0.1 (1) |
| R49 | 0.0 (0) | 0.0 (0) | 0.6 (1) | 0.0 (0) | 0.0 (0) | 0.1 (1) |
| R50 | 0.0 (0) | 0.0 (0) | 1.1 (2) | 0.0 (0) | 0.0 (0) | 0.2 (2) |
| R51 | 0.0 (0) | 0.0 (0) | 0.0 (0) | 0.0 (0) | 0.6 (1) | 0.1 (1) |
| R52 | 0.6 (1) | 0.0 (0) | 0.0 (0) | 0.0 (0) | 0.0 (0) | 0.1 (1) |
| Total | 100.0 (180) | 100.0 (180) | 100.0 (180) | 100.0 (180) | 100.0 (180) | 100.0 (900) |

N= number of REP profiles
