# Supplementary material for: The genetic diversity of commensal Escherichia coli strains isolated from non-antimicrobial treated pigs varies according to age group
Source: PLoS One. 2017 May 30;12(5):e0178623. doi: 10.1371/journal.pone.0178623 (PMC5448805; doi:10.1371/journal.pone.0178623)
Supplement: S3 Table — (DOCX) [file pone.0178623.s006.docx]

**Table S3.** PFGE patterns generated by the *E. coli* strains assigned to different REP profiles.

| **REP profiles** | **PFGE patterns** |
| --- | --- |
| R1 | X1, X39, X40, X41, X44, X57, X67 |
| R2 | X2 |
| R3 | X3 |
| R4 | X4 |
| R5 | X5 |
| R6 | X6 |
| R7 | X7, X33, X40, X42, X48 |
| R8 | X8, X53, X64 |
| R9 | X9 |
| R10 | X10, X64 |
| R11 | X11 |
| R12 | X12 |
| R13 | X13 |
| R14 | X14 |
| R15 | X15 |
| R16 | X16, X56 |
| R17 | X4 |
| R18 | X17 |
| R19 | X18 |
| R20 | X19 |
| R21 | X20, X26, X38 |
| R22 | X21 |
| R23 | X22 |
| R24 | X23, X34, X41 |
| R25 | X24, X37 |
| R26 | X25 |
| R27 | X27 |
| R28 | X28, X43, X48, X54 |
| R29 | X29 |
| R30 | X30 |
| R31 | X32 |
| R32 | X43 |
| R33 | X45 |
| R34 | X46 |
| R35 | X47 |
| R36 | X49 |
| R37 | X50 |
| R38 | X51 |
| R39 | X55 |
| R40 | X52 |
| R41 | X58 |
| R42 | X59 |
| R43 | X60 |
| R44 | X62 |
| R45 | X36, X36 |
| R46 | X35 |
| R47 | X63, X64 |
| R48 | X65 |
| R49 | X63 |
| R50 | X66 |
| R51 | X61 |
| R52 | X31 |
